# Radiation dosimetry performance appraisal of the optimal concentration of nitro blue tetrazolium dye in PVA composite film at low X-ray doses

**DOI:** 10.1016/j.heliyon.2024.e41319

**Published:** 2024-12-18

**Authors:** Reham Albejadi, Saleh Alashrah, Yassine El-Ghoul, Zabih Ullah, Saleh A. Almatroodi

**Affiliations:** a Department of Physics, College of Science, Qassim University, Buraidah 51452, Saudi Arabia; b Department of Chemistry, College of Science, Qassim University, Buraidah 51452, Saudi Arabia; c Department of Pharmaceutical Sciences, College of Dentistry and Pharmacy, Buraydah Colleges, Alqassim, Saudi Arabia; d Department of Medical Laboratories, College of Applied Medical Sciences, Qassim University, Buraydah 51452, Saudi Arabia

**Keywords:** Nitro blue tetrazolium, Polymer, ImageJ, CIELab, X-ray diffraction, Scanning electron microscopy

## Abstract

**Purpose:**

Due to the extensive use of radiation in various fields, such as food safety, sterilizing surgical materials, and medical diagnostics, it is essential to minimize radiation exposure for both patients and healthcare professionals, even at low doses. To meet this requirement, a composite film has been developed using polyvinyl alcohol (PVA) polymer and nitro blue tetrazolium (NBT) dye to measure low radiation doses effectively.

**Methods:**

Various concentrations of NBT dye (ranging from 0.75 to 15 g/L) were tested to determine the optimal concentration for maximum efficiency. The films were exposed to low doses of X-rays ranging from 20 to 100 mGy.

**Results:**

The PVA/NBT films exhibited good stability for 30 days after irradiation when stored in darkness. UV–visible absorption spectra showed a peak at 376 nm, shifting to 384 nm after exposure to X-rays. Furthermore, the response curve based on UV–Vis absorptions revealed a linear increase in absorbance with increased radiation doses up to 60 mGy (R = 0.99). XRD analysis indicated significant structural rearrangements in films with higher NBT concentrations and radiation doses. Specifically, X-ray irradiation caused two distinct peaks to shift from 2θ = 20.12° and 40.18° to 2θ = 19.66° and 41.24°. SEM analysis revealed that exposure to increased radiation doses resulted in significant morphological changes in PVA/NBT films, resembling torn tree trunks, especially with higher NBT concentrations. The preparation method was validated by XRF analysis, and the solubility of the NBT salt was excellent, with an R^2^ value of 0.9656. Results from Spectro colorimetric studies indicated that different CIEL∗a∗b∗ coordinates, as well as the total color difference (ΔE∗_ab_) and color strength (K/S), were directly proportional to the applied dose intensity, correlating with increasing NBT concentration.

**Conclusion:**

The PVA/NBT dosimeter containing 10 g/L of NBT shows promising potential as an effective alternative for the prompt and accurate detection of low X-ray doses in diagnostic radiology.

## Graphical abstract

**Figure:**
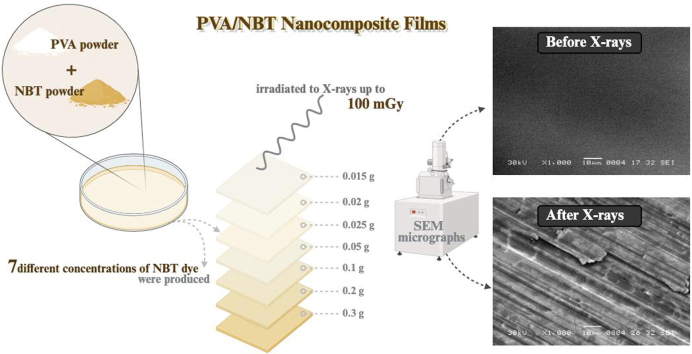

## 1 Introduction

Radiation technique treatment is extensively applied in different fields involving routine dosimetry applications. These applied fields include Water remedy, food irradiation, polymerization, nanotechnology, medical sterilization, and diagnostic radiology [[1], [2], [3], [4], [5]]. Different levels of radiation dose are investigated. The variation of the dose intensity classified as high, medium, or low specifies the field of application of the radiation. Currently, the accurate detection and assessment of the delivered radiation dose represent an essential requirement in many applications. The main challenge still exists today with low doses given the lack of appropriate dosimeters in this low range [[6], [7]]. The more significant solution widely reported was the use of different dosimetric films.

One of the most widely used film dosimeters in radiation physics applications for over 20 years is the Radiochromic film (RCF). RCF films are dosimeters whose crystalline-sensitive elements undergo structural changes when exposed to ionizing radiation. The interaction between ionizing radiation and the film's monomer leads to a polymerization process within the sensitive element, resulting in a visible change in the film's color corresponding to the radiation dose [8].

RCF films offer numerous advantages, including relatively low energy dependence and high spatial resolution. Importantly, RCFs are tissue-equivalent and can measure a wide range of doses (from 10^−2^ to 10^6^ Gy). Furthermore, they are insensitive to visible light and do not require physical or chemical processing. In contrast, radiographic films necessitate wet chemical processing and are sensitive to room light [[9], [10]]. Recently,

The film can be produced using a polymer matrix doped with a dye as an indicator [11]. Various polymer materials have been utilized for dosimetry applications, such as polyvinyl alcohol (PVA) [12], polyvinyl butyral (PVB) [13], polyvinylidene fluoride (PVDF) [14], and polyvinyl chloride (PVC), polypropylene [15]. Similarly, indicators investigated in the literature include nitro blue tetrazolium chloride (NBT) [16], cresol red (CR) [17], tetrazolium violet (TV) [18], and leuco crystal violet (LCV) [13].

In this context, a film based on a combination of PVA as a matrix and NBT dye has been studied extensively due to the favorable properties of these materials. PVA is characterized as a versatile, semi-crystalline polymer with excellent chemical and physical properties attributed to the presence of OH groups and the ability to form hydrogen bonds [19]. Notably, PVA offers superior flexibility and water solubility, making it safe, stable, and suitable for biomedical applications across various radiation doses [20]. Additionally, its non-toxic nature further enhances its suitability for use in this research [21].

Tetrazolium salts are heterocyclic organic compounds that undergo a vivid color change and form water-insoluble formazans upon exposure to radiation [8]. Nitro blue tetrazolium (NBT) is a di-tetrazolium salt known for its dramatic color change upon radiation exposure, transitioning from reduced mono-formazan (MF^+^) to di-formazan (DF) with increasing radiation doses, making it ideal for dosimetry applications [[22], [23]].

In one experiment, a PVA/NBT film exposed to low X-ray doses was examined. Various aspects were studied, including film stability, CIEL∗a∗b∗ analysis, and FTIR analysis. UV–visible absorption analysis revealed peaks at 430 nm and 450 nm for higher doses. The film's color changed from light yellow to yellowish-brown post X-ray exposure. Alashrah et al. concluded that PVA/NBT films can be used effectively in routine diagnostic radiology within the 2–20 mGy range [24].

The same group also investigated the impact of adding silver nitrate (AgNO_3_) to PVA/NBT films [25]. The CIEL∗a∗b∗ parameters, color difference (ΔE∗_ab_), and color strength (K/S) showed linear increases with X-ray exposure. XRD results of PVA/Ag^+^/NBT demonstrated significant structural modifications due to crystallinity changes post-irradiation. SEM micrographs confirmed structural changes within the film following X-ray exposure, indicating the potential use of PVA/Ag^+^/NBT films for routine medical dosimetry within the 2–20 mGy range.

In another study, Rabaeh et al. prepared PVA/NBT films with varying concentrations of NBT dye (1, 1.5, and 5 mM) exposed to doses ranging from 5 to 50 kGy [26]. They observed that increasing NBT dye concentration enhanced the film's dose-response. Furthermore, the addition of sodium formate and Triton X-100 positively affected film performance. Parameters such as temperature, humidity, stability, and others were evaluated, highlighting the film's utility in routine dosimetry within industrial high-dose ranges.

In the current study, a PVA/NBT film was developed by preparing a diverse range of NBT concentrations to enhance the film's sensitivity. The primary objective of this study is to determine the optimal NBT concentration in PVA/NBT films under specified working conditions and to assess the effectiveness of the films at low X-ray doses in the mGy scale. The films were evaluated using colorimetric analysis based on the CIEL∗a∗b∗ system and the color strength (K/S) test. Additionally, ImageJ software and UV–visible absorption spectra were employed for further analysis. Structural and morphological techniques, including X-ray diffraction (XRD), X-ray fluorescence (XRF), and scanning electron microscopy (SEM), were utilized to highlight the effectiveness of these films.

## 2 Experimental

### 2.1 Materials

The nitro blue tetrazolium chloride NBT (C_40_H_30_Cl_2_N_10_O_6_; M_W_ = 817.65; +98 %) of Alfa Aesar brand was purchased from Thermo Fisher Scientific (USA) for use as a dye indicator of the film, while the mixture polymer of films was polyvinyl alcohol (PVA, M_W_ = 85–124 kDa with a hydrolysis degree of 85–90\%), was acquired from Sigma Aldrich (St. Louis, MO, USA). Ultrapure water (Milli-Q® Direct, Darmstadt, Germany), was used in the solubilization process during the preparation of films.

### 2.2 Preparation of thin films

Seven samples were prepared by dissolving 2 g of polyvinyl alcohol in 20 ml of distilled water for each sample. To ensure homogeneity, the solutions were stirred for 2 h at a constant temperature of 80 °C and then allowed to cool to room temperature. Nitro blue tetrazolium (NBT) indicator solutions were prepared at seven different concentrations (ranging from 0.75 to 15 g/L) by dissolving varying amounts of NBT in distilled water. Each NBT mixture was then added to the respective PVA solution and stirred for 1 h at room temperature. Subsequently, each solution was poured onto a glass Petri dish and left to dry in darkness at room temperature for two days. The transparent thin films in the Petri dishes were peeled away uniformly after drying. Using a thickness gauge, the average thickness of the produced films was found to be approximately 100 μm. Finally, the dried films were cut into small 2 cm squares and stored in airtight envelopes to protect them from light until they were ready for irradiation.

Finally, the films were irradiated using a digital X-ray fluoroscopic machine (GE, healthcare, model Al01CII, Chicago, USA) with five different doses of 20–100 mGy. The parameters of the exposure technique were adjusted (kVp = 60, mAs = 16 and at 100 cm) to produce the exact exposure doses.

### 2.3 Characterization and measurement of films

In this study, ImageJ software was utilized to assess the stability of the films and to measure the relative density of the red channel by scanning the PVA/NBT films using a Canon (CanoScan LiDE 110) scanner. Digital images of the scanned films were saved in TIFF format at a resolution of 300 dpi. Each film was measured within an area of 0.028 square inches, which provided sufficient statistical data despite the small size of the films. Relative density was calculated by subtracting the pixel value of the unirradiated film from the irradiated film, and the results were then normalized. Percentage relative density was plotted against different concentrations.

Furthermore, colorimetric analyses of irradiated and non-irradiated films were conducted using a 3NH YD5010 spectrodensitometer in the visible spectrum. The measurements were performed under specific conditions including a standard observer angle of 10°, daylight standard illuminant D65, and M0 measurement condition. The SDQC program, recommended by the manufacturer, was employed via a USB connection with the spectrodensitometer to measure CIEL∗a∗b∗ values and total color difference (ΔE∗_ab_). Additionally, color strength was assessed by measuring film reflectance at wavelengths ranging from 480 to 700 nm using the Senior Mode (densitometer). All variables were plotted as functions of different NBT concentrations using Microsoft Excel.

The concentration of elements present in PVA/NBT films was determined using an ARL^TM^Quant’X EDXRF spectrometer manufactured by Thermo Scientific™ Inc., USA. The spectrometer's x-ray tube operates at voltages ranging from 4 to 50 kV, with a current of 0.02–1.98 mA, and uses rhodium (_45_Rh) as its anode material. The released X-rays are detected by ARL Quant’X's Si(Li) semiconductor detector, which has a crystal area of 15 mm^2^ and a crystal depth of 3.5 mm. The filters utilized are shown in Table 1. Microsoft Excel was used to analyze the outcomes.

**Table 1:** List filters of ARL Quant’X EDXRF spectrometer [27].

Filter	Voltage (kV)	Live time (s)	Elements
Cu thick	50	240	Sn, Sb
Cu thin	50	800	Mo, Cd
Pd thick	30	1,600	As, Br, Sr, Pb
Pd medium	20	960	Cu, Zn
Pd thin	16	600	Fe, Co, Ni, Mn
Aluminum	12	1,000	Ti, V, Cr
Cellulose	8	200	S, Cl, K, Ca
No filter	4	100	Mg, Al, Si

The UV–visible absorption spectra of the PVA/NBT films were measured using a Shimadzu UV-2501PC spectrophotometer from Kyoto, Japan. The measurements were taken across a wavelength range of 200–600 nm.

X-ray diffraction patterns were obtained using the Rigaku Ultima IV X-ray Diffractometer. The samples were recorded with a step width of 0.02° at the 2θ angle, ranging from 10° to 90°. The instrument was operated at a voltage of 40 kV and a current intensity of 40 mA. The resulting 2θ values were plotted against their respective intensities for different doses using Matlab software.

Micrographs of specific films were captured using the JEOL scanning electron microscope model JSM-5500 from Akishima, Tokyo, Japan, to study the impact of radiation on film surface morphology. The measurements were conducted at 30 kV acceleration voltage and 1000× magnification. To improve the surface conductivity of the samples, a thin layer of gold (Au) was applied before measuring them using the JFC-1300 auto fine coater from the JEOL brand.

## 3 Results and discussion

### 3.1 Stability of PVA/NBT film dosimeter after irradiation

**Fig. 1:**
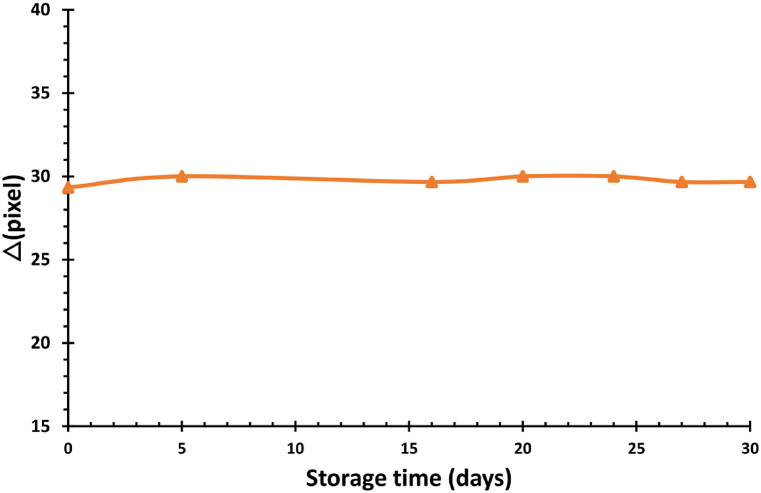
Stability of PVA/NBT film containing 5 g/L of NBT stored in the dark.

PVA/NBT film containing 5 g/L of NBT underwent stability evaluation after exposure to 80 mGy of X-rays. The red color channel of the irradiated film was compared to the control film using ImageJ software. Following the irradiation, the film was stored under standard laboratory conditions in the dark for 30 days. The study determined that there were no significant changes in the film's stability over the test period. These findings were consistent with previous research conducted by Ref. [24]. Fig. 1 displays the film's good stability at various time intervals after the X-ray irradiation.

### 3.2 The color change of PVA/NBT films after irradiation

**Fig. 2:**
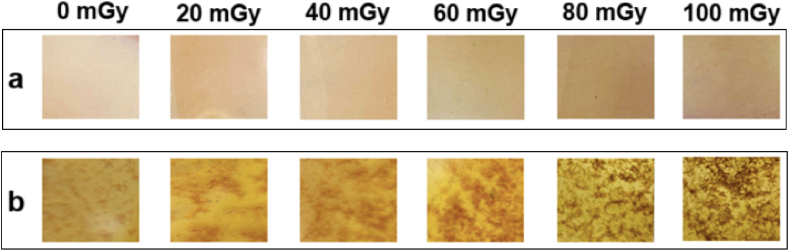
Color change in PVA/NBT films for two different concentrations of NBT dye a: 1 g/L, b: 10 g/L, after being exposed to various X-ray doses.

The PVA/NBT composite films exhibited a color change from light yellow to yellowish-brown when exposed to low doses of X-rays. Fig. 2 illustrates two samples of the films with different concentrations of NBT dye: one containing 1 g/L and the other containing 10 g/L. Increasing the concentration of NBT dye resulted in the films appearing more yellowish, while higher X-ray doses led to darker films across all concentrations, albeit with varying intensities. Films with higher concentrations of NBT showed a more pronounced color change after irradiation, particularly those containing 10 g/L of NBT. This heightened responsiveness can be attributed to the increased amount of NBT dye present, which enhances the reduction of NBT^+2^ upon irradiation, leading to the formation of mono-formazan and di-formazan [[28], [29]]. The films exhibited exceptional sensitivity to X-rays, even at low doses, making them well-suited for diagnostic dosimetry. These changes were easily discernible without the need for specialized equipment, demonstrating their effectiveness in this field.

### 3.3 Relative density

**Fig. 3:**
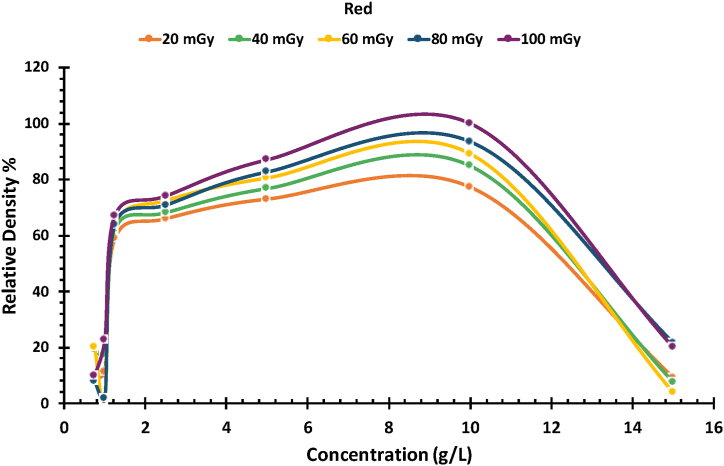
Relative density of PVA/NBT film dosimeters containing different concentrations of for the red channel.

The relative density of PVA/NBT films, irradiated by low doses of X-rays, was examined using ImageJ software. The software can measure the RGB colors of digital images, but in this study, only the red color channel was adopted as it showed the most significant variation in the obtained values. Concentrations below 1.25 g/L and above 10 g/L were found to be ineffective for low X-ray doses. In contrast, the PVA/NBT films with NBT compositions from 2.5 to 10 g/L showed a positive response, as illustrated in Fig. 3. Fig. 3 shows a linear increase in redness as the concentration of NBT dye increases. The relative density increases with the high X-ray dose until it reaches an NBT concentration of 10 g/L. After that point, the relative density gradually decreases with increasing concentration. According to ImageJ analysis, the concentration range of NBT dye suitable for PVA/NBT films is between 2.5 and 10 g/L.

### 3.4 Color strength K/S

The Kubelka-Munk equation is one of the most common equations used to relate reflectance to dye concentration [30]. It has been utilized in various studies, including dosimetry [[24], [25]] and evaluating the effect of dyes on fabrics [[31], [32]]. The K-M equation is expressed in terms of absorption coefficient (K) and scattering coefficient (S), and it is given by Equation (1).

(1) $$K/S=\frac{{\left(1-R\right)}^{2}}{2R}$$

Where R is the reflectance at wavelength λ.

**Fig. 4:**
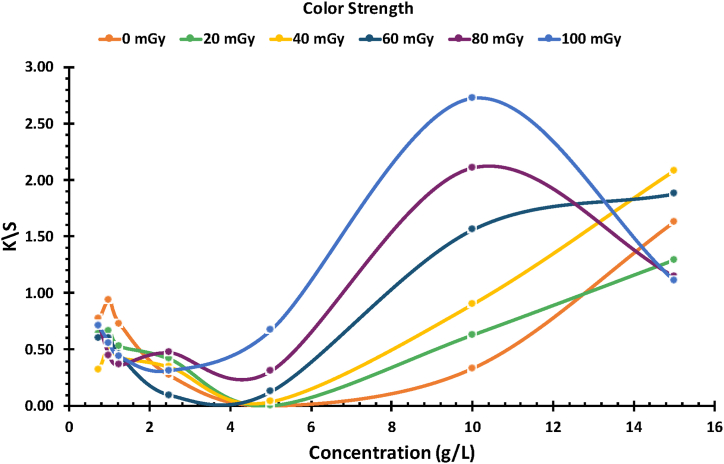
Color strength K/S of PVA/NBT films after exposure to varying X-ray doses at different NBT concentrations.

In Fig. 4, the graph illustrates K/S values corresponding to the concentration of NBT dye in PVA/NBT films at different X-ray doses, For unirradiated films, a linear increase in K/S values was observed as the concentration of NBT dye increased from 5 to 15 g/L, resulting in films exhibiting a yellow color with higher NBT concentrations. However, with increased X-ray doses, each concentration exhibited distinct behavior, except for concentrations of 5 and 10 g/L. At these concentrations, there was a linear increase in K/S with increasing doses, leading to a gradual darkening of the films. Fig. 4 highlights those concentrations higher than 10 g/L showed a disrupted response. The results indicate a significant increase in the dose-response of PVA/NBT films as the concentration of NBT dye increases, consistent with findings from a previous study [26]. The primary objective of this study was to identify the optimal concentration of NBT that exhibits the best performance based on dosage. It was observed that at a concentration of 10 g/L, the films displayed the highest sensitivity to X-rays compared to other concentrations.

### 3.5 Colorimetric study

The CIEL∗a∗b∗ system, which was recommended for the first time in 1976, is widely used to specify colors and color differences. It consists of three parameters: L∗, a∗, and b∗. L∗ represents lightness, with a value of 0 indicating perfect black and 100 indicating perfect white. Positive values of a∗ indicate red color, while negative values represent green color. Similarly, b∗ represents yellow color for positive values and blue for negative values [33]. These parameters were obtained directly using a spectrodensitometer. However, Equation (2) was used to calculate (ΔE∗_ab_), which represents color difference.

(2) $${\Delta E}_{ab}^{*}=\sqrt{{\left({\Delta L}^{*}\right)}^{2}+{\left({\Delta a}^{*}\right)}^{2}+{\left({\Delta b}^{*}\right)}^{2}}$$

ΔL∗ = L∗_1_ − L∗_0_, where L∗_1_ is the value for irradiated film, and L∗_0_ is the value for non-irradiated film, similarly for Δa∗ and Δb∗.

**Fig. 5:**
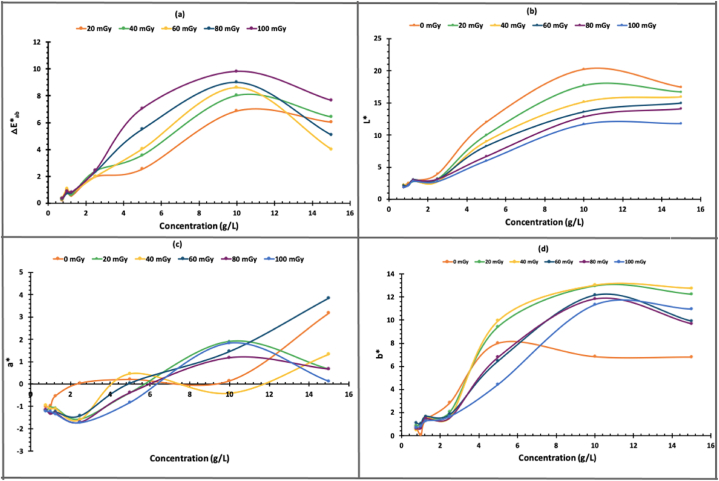
The CIEL∗a∗b∗ parameters and total color difference ΔE∗_ab_ for PVA/NBT films at different NBT concentrations, exposed to different X-ray doses. Where **(a)** represents ΔE∗_ab_, **(b)** L∗, **(c)** a∗, and **(d)** b∗.

In Fig. 5a, we can observe that ΔE∗_ab_ increased in a linear manner as the concentration of NBT increased up to 10 g/L. However, after reaching this concentration, the value of ΔE∗_ab_ decreased. It is worth noting that only concentrations of 5 and 10 g/L demonstrated a clear response to increasing X-ray doses applied to the films. Beyond a concentration of 10 g/L, the color change was not very noticeable, and the film became too dark. Therefore, it was not possible to observe the impact of X-rays on the film directly. The L∗ results (Fig. 5b) exhibit a pattern similar to that of ΔE∗_ab_ where L∗ values increase significantly with increasing NBT concentration up to 10 g/L and then decrease. However, L∗ values decrease as X-ray doses increase, as the films become darker after irradiation, in line with published literature [[34], [35]]. According to Fig. 5c, there was a linear increase in the amount of red color (+a∗) as the concentration of NBT increased, regardless of the order of dosing. Similarly, Fig. 5d indicated a linear increase in yellow (+b∗) color up to 10 g/L, beyond which it reaches a saturation point. The aforementioned results demonstrate that PVA/NBT film with NBT concentrations of 5 and 10 g/L can serve as reliable dosimeters to detect low doses of X-ray exposure.

### 3.6 UV–visible absorption spectra

**Fig. 6:**
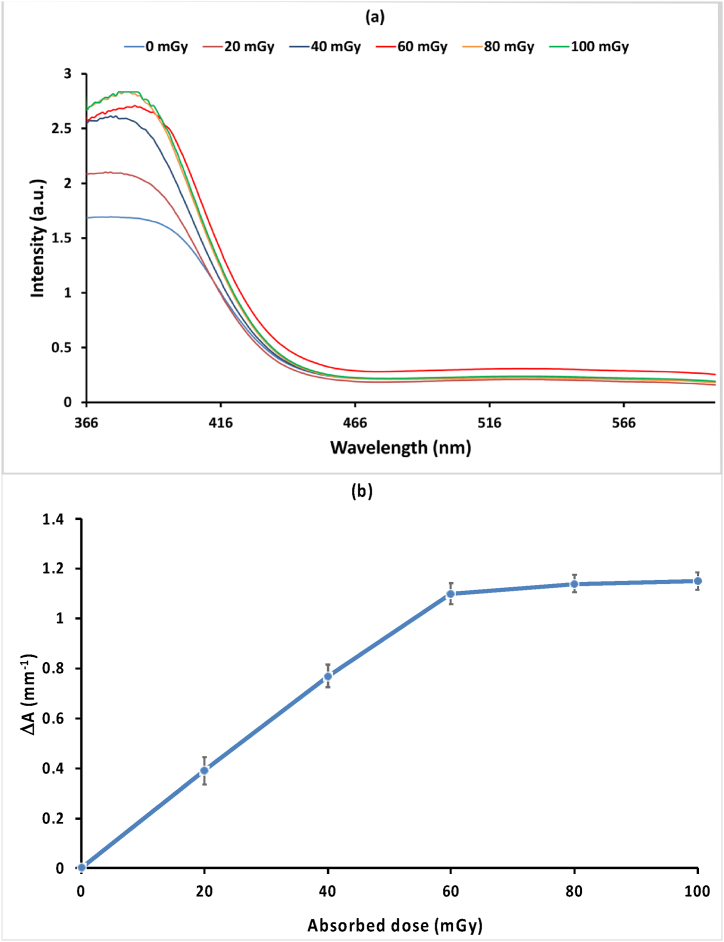
(a) Represents the UV–Visible absorption spectra of a PVA/NBT film containing 10 g/L of NBT dye before and after exposure to varying doses of X-rays, while **(b)** shows the dose-response curve of the change in absorbance per unit thickness as a function.

The UV–Visible absorption spectra of a PVA/NBT film incorporating 1 and 10 g/L of NBT dye were recorded across the wavelength range of 200–600 nm. Fig. 6a illustrates the spectrum at different X-ray doses, where the absorption peak initially appeared at 376 nm and shifted to 384 nm at a dose of 60 mGy after X-irradiation. The intensity of these bands progressively increased with higher applied X-ray doses. The study indicates that only NBT dye concentrations of 1 g/L and below are suitable for UV-absorption analysis. Higher concentrations did not yield meaningful results due to the high absorbance values caused by the dark color of the films. Regarding the change in absorbance per unit thickness **(Δ**A.mm^−1^). Fig. 6b presents the dose-response curve of the same sample at 376 nm. The results show an increase in absorbance with increasing X-ray doses up to 60 mGy, beyond which no significant change was observed (stability zone). This stabilization could be attributed to the low amount of NBT dye present in the film.

### 3.7 X-ray fluorescence study

**Fig. 7:**
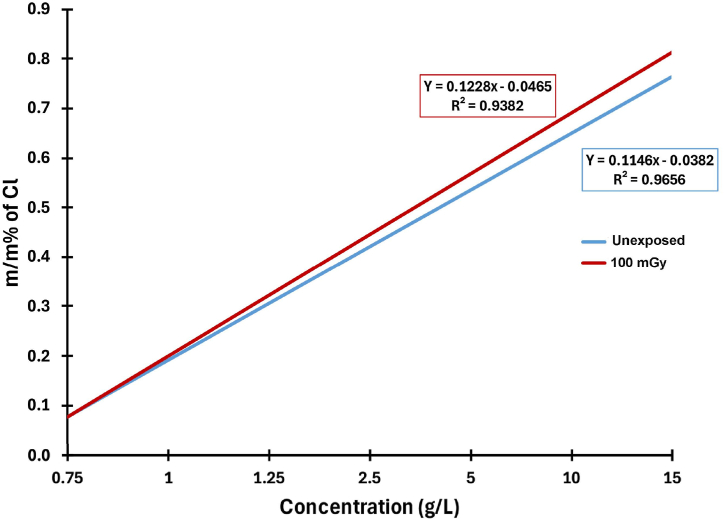
The mass percent (m/m%) of Cl element against NBT concentration amounts in PVA/NBT films for unexposed and irradiated films.

The chemical composition of PVA/NBT films before and after X-ray irradiation was examined using X-ray fluorescence (XRF) analysis. This method was chosen for its ability to provide precise and detailed information about the elements present in the films. The study specifically focused on the element chlorine (Cl) within the PVA/NBT composite films, as it is the element most significantly impacted by radiation. The graph in Fig. 7 shows the mass percentage (m/m%) of Cl for unexposed and irradiated films at 100 mGy as a function of NBT concentration. The results indicated that the concentration of chlorine in PVA/NBT films is directly proportional to the amount of NBT used, which was an expected outcome. This uniform increase in unexposed films serves as evidence of the effectiveness of the NBT salt dissolution method used, with a linear regression coefficient of R^2^ = 0.9656. However, after irradiation, a slight increase in Cl levels was observed in PVA/NBT films at NBT concentrations above 1.25 g/L. Conversely, no significant effect was observed at concentrations below 1.25 g/L. At the highest concentration of 15 g/L, the results showed a correspondence with values of 10 g/L, indicating that saturation had occurred. The findings suggested that the most effective concentration range for NBT lies between 1.25 and 10 g/L. The study also revealed that the increase in Cl concentration within this concentration range indicates the response to radiation.

### 3.8 X-ray diffraction study

**Fig. 8:**
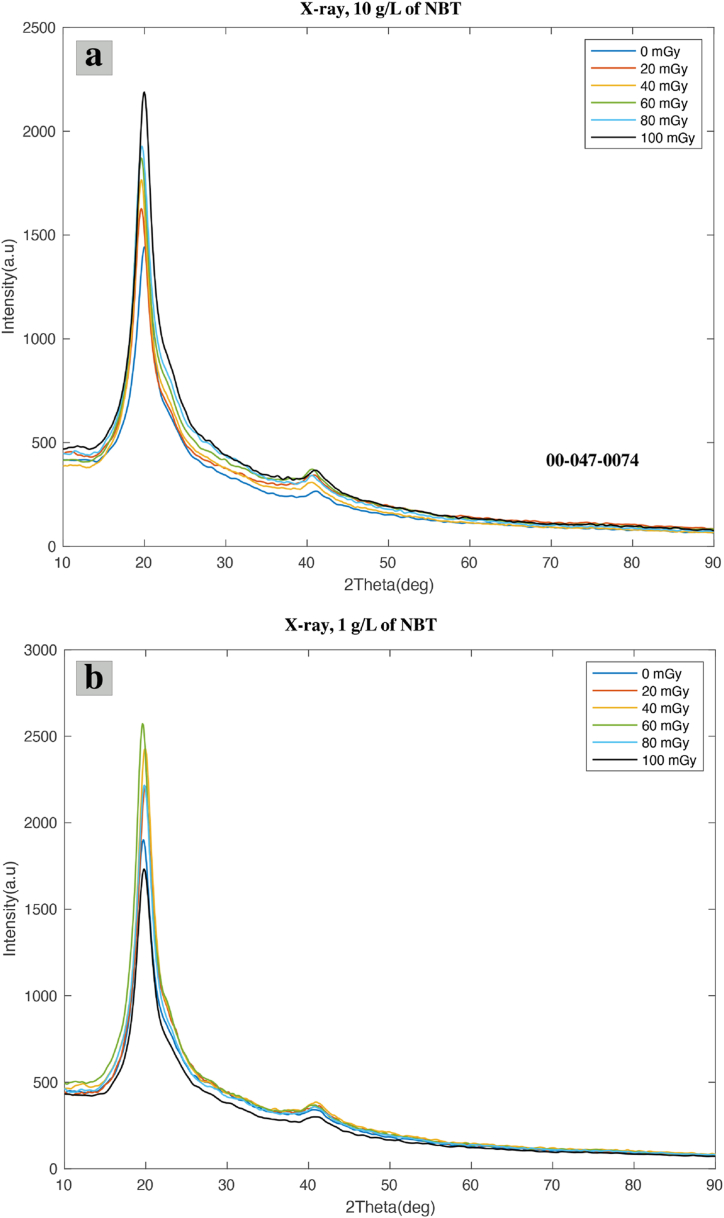
The XRD patterns of PVA/NBT films containing **(a)** 10 g/L and **(b)** 1 g/L of NBT dye at different exposure doses**.**

X-ray diffraction has always been reported as an effective technique to study various structural changes following exposure to different types of irradiation sources [[36], [37]]. The impact of X-rays on the structural and crystalline properties of composite films composed of PVA/NBT was analyzed through XRD patterns. Two samples containing different amounts of NBT dye (1 and 10 g/L) were exposed to varying doses of X-rays and examined before and after, as depicted in Fig. 8. Upon analyzing the diffractograms of both films, it was observed that they exhibited two distinct peaks. The first strong characteristic peak was observed at 2θ = 19.82° and 20.12°, respectively, for the 1 and 10 g/L samples of NBT. These results suggest the semi-crystalline nature of pure PVA [38], corresponding to the reflection plane of (101) [39]. Additionally, a minor peak at 2θ = 40.36° and 40.94° was observed for the 1 and 10 g/L samples of NBT, respectively. These peaks correspond to the (111) reflection plane [40] and confirm the crystalline phase of PVA [41]. Both concentrations showed a shift in peak position after exposure to X-rays, as detailed in Table 2. At a concentration of 10 g/L, the minor peak shifted from 40.18° to 41.24° after exposure to X-rays at 20–100 mGy, while the main peak shifted from 20.12° to 19.66° at 80 mGy. Furthermore, the higher concentration exhibited a consistent increase in peak intensity with increasing applied X-ray doses, showing a noticeable distinction between the dose curves. Conversely, the lower concentration did not demonstrate this difference. These results confirm that after irradiation, the PVA/NBT films underwent structural rearrangement, evidenced by shifts in peak position and changes in intensity. The gradual structural change with the absorbed dose is related to the reduction of NBT^2+^ to mono-formazan (MF^+^) then to a stable hydrophobic di-formazan (DF) structure under the effect of the applied irradiation [28,29]. These findings align with previous research on PVA films doped with other substances and exposed to high doses of electromagnetic radiation [34,[42], [43]] indicating that low X-ray irradiation produces similar effects as high doses of radiation.

**Table 2:** A list of principal and minor peak values for PVA/NBT films containing 1 and 10 g/L of NBT, exposed to varying X-ray doses.

Dose(mGy)	10 g/L of NBT		1 g/L of NBT	
	2θ (degree)	Intensity (a.u)	2θ (degree)	Intensity (a.u)
0	20.12	1,548	19.82	1,999
	40.94	303	40.36	388
20	19.82	1,699	20.12	2,265
	40.18	385	41.28	398
40	19.62	1,786	19.86	2,534
	40.38	328	41.86	428
60	19.64	2,001	19.66	2,671
	40.42	418	40.10	410
80	19.66	2,026	19.68	2,298
	41.79	372	40.56	387
100	20.00	2,266	19.58	1813
	41.24	432	40.98	338

### 3.9 SEM morphological analysis

**Fig. 9:**
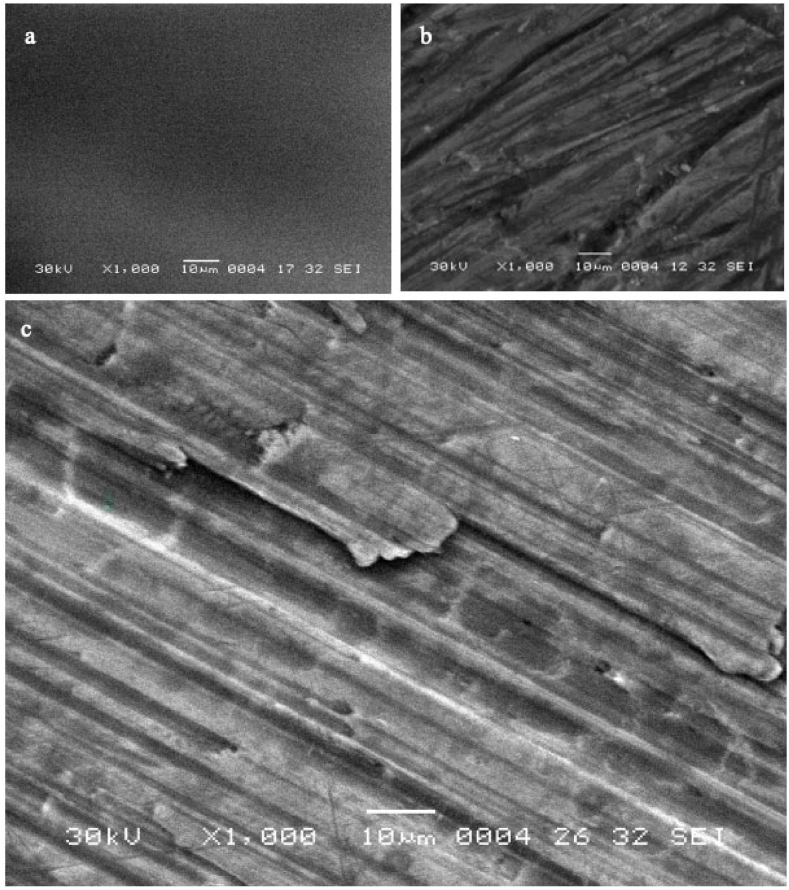
SEM micrographs of unirradiated PVA/NBT film with 1 g/L NBT concentration **(a)**, and irradiated films with 80 mGy at a concentration of NBT 1 g/L **(b)** and 10 g/L **(c)**.

SEM analysis was conducted on samples of PVA/NBT films with different concentrations to investigate the impact of low X-ray doses on their surface morphology and microstructure. Fig. 9a shows the unexposed PVA/NBT film, which has a smooth and homogeneous surface without any cracks. However, when the films were exposed to X-rays (Fig. 9b), several cracks appeared, and the surface structure changed, resembling torn tree trunks, with an increase in the applied X-ray doses. Fig. 9c indicates that as the concentration of the NBT dye increased, the number of cracks also increased, and they became more apparent for the same doses of X-rays used. This suggests that the PVA/NBT film is sensitive to low doses of X-rays for higher NBT concentrations, making it an effective dosimeter in diagnostic applications. This technique has been previously employed on PVA films with other compounds and has yielded similar results before and after exposure to radiation [[44], [45]], as well as in low doses of X-ray [46].

## 4 Conclusion

The PVA/NBT composite film, developed with varying concentrations of NBT dye ranging from 0.75 to 15 g/L, exhibited remarkable sensitivity to low doses of X-rays, a discovery that could pave the way for new applications in diagnostic radiology. This sensitivity was validated through chemical, physical, and colorimetric analyses, revealing that it increased with the concentration of NBT dye. Upon exposure to X-rays, the films changed to a yellowish-brown hue, with the intensity of the color deepening as the dosage increased. This intriguing correlation between the NBT dye concentration and the film’s responsiveness to X-rays was further supported by SEM analysis, which showed several cracks had appeared and changes in surface structure. XRD pattern analysis confirmed structural rearrangement after irradiation, particularly noticeable at higher NBT concentrations. The shift of two distinct peaks from 2θ = 20.12° and 40.18° to 2θ = 19.66° and 41.24° after irradiation further underscores the sensitivity of the films to X-rays, even at low doses. Moreover, the PVA/NBT films demonstrated high stability even after being irradiated for up to 30 days in the dark. XRF analysis confirmed the effectiveness of the preparation method for concentrations ranging from 1.25 to 10 g/L. The dose-response curve from UV–Vis absorptions revealed that the PVA/NBT film exhibited high sensitivity to low X-ray doses, even with low NBT dye concentrations. Furthermore, utilizing the CIEL∗a∗b∗ system, K/S color strength test and ImageJ analysis enabled a comprehensive colorimetric study, all of which indicated increased values as NBT concentration rose. This aligns with the observable color change following radiation exposure. Based on the outcomes of these various tests, NBT concentrations between 2.5 and 10 g/L are recommended for optimal results. Specifically, a concentration of 10 g/L proved to be the most X-ray-sensitive within the low dose range (in the mGy scale), making it ideal for use in diagnostic radiology.

In future research, it is crucial to delve deeper into the non-response behavior of PVA/NBT films with high concentrations of NBT dye. It is also important to conduct experiments on films with different concentrations exposed to various types of radiation in order to broaden and enhance our understanding of their properties.

## CRediT authorship contribution statement

**Reham Albejadi:** Writing – review & editing, Writing – original draft, Visualization, Validation, Software, Resources, Project administration, Methodology, Investigation, Funding acquisition, Formal analysis, Data curation, Conceptualization. **Saleh Alashrah:** Writing – review & editing, Writing – original draft, Visualization, Validation, Supervision, Resources, Project administration, Methodology, Investigation, Formal analysis, Data curation, Conceptualization. **Yassine El-Ghoul:** Writing – review & editing, Writing – original draft, Visualization, Validation, Methodology, Investigation, Formal analysis. **Zabih Ullah:** Writing – review & editing. **Saleh A. Almatroodi:** Resources.

## Ethical considerations

Ethical approval is not applicable for this study.

## Data availability statement

No data was used for the research described in the article.

## Funding

The authors declare that no funds, grants, or other support were received during the preparation of this manuscript.

## Declaration of Competing Interest

The authors declare that they have no known competing financial interests or personal relationships that could have appeared to influence the work reported in this paper.

## Figures and Tables

**Fig. 10:**
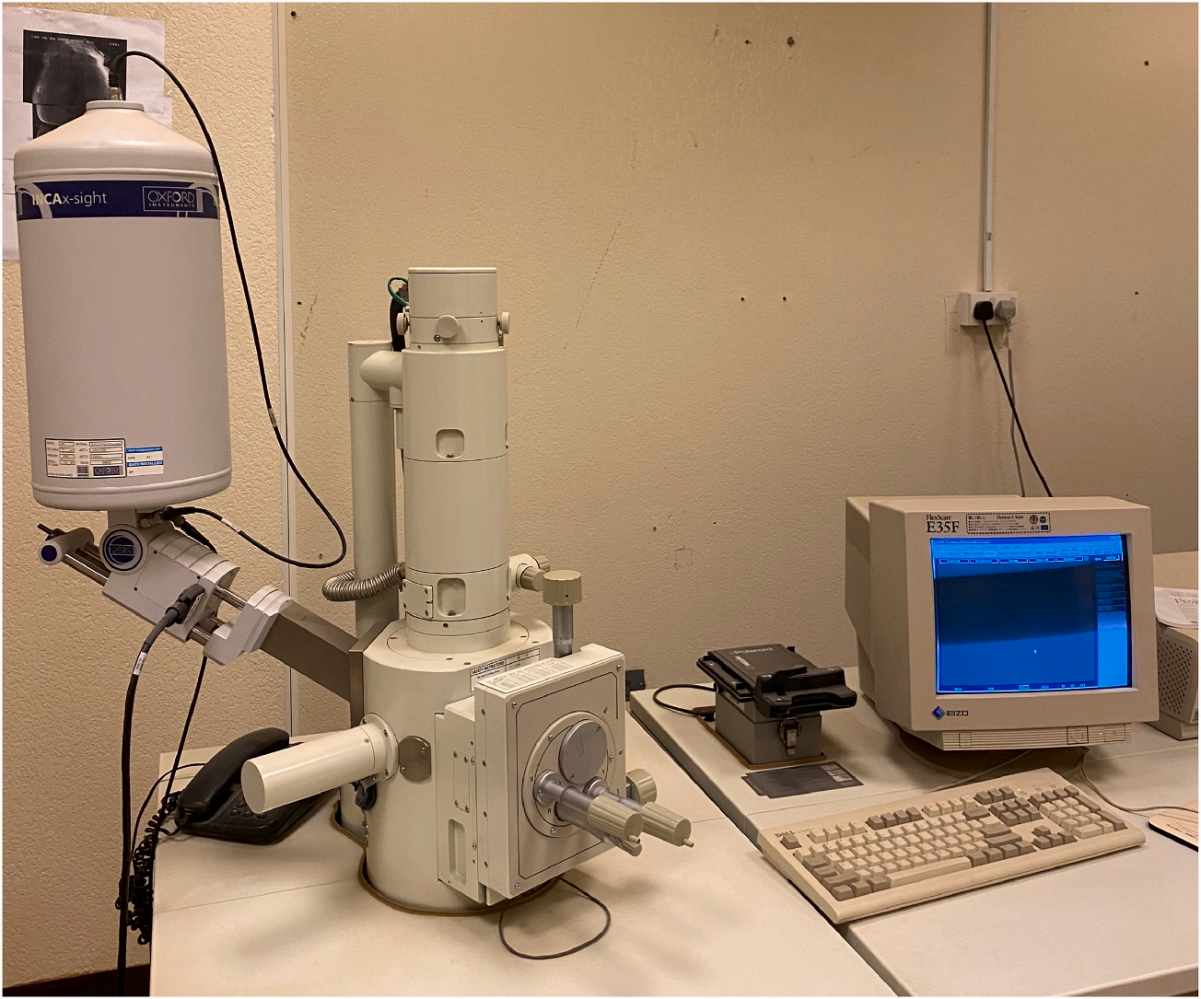
Scanning electron microscope used.

**Fig. 11:**
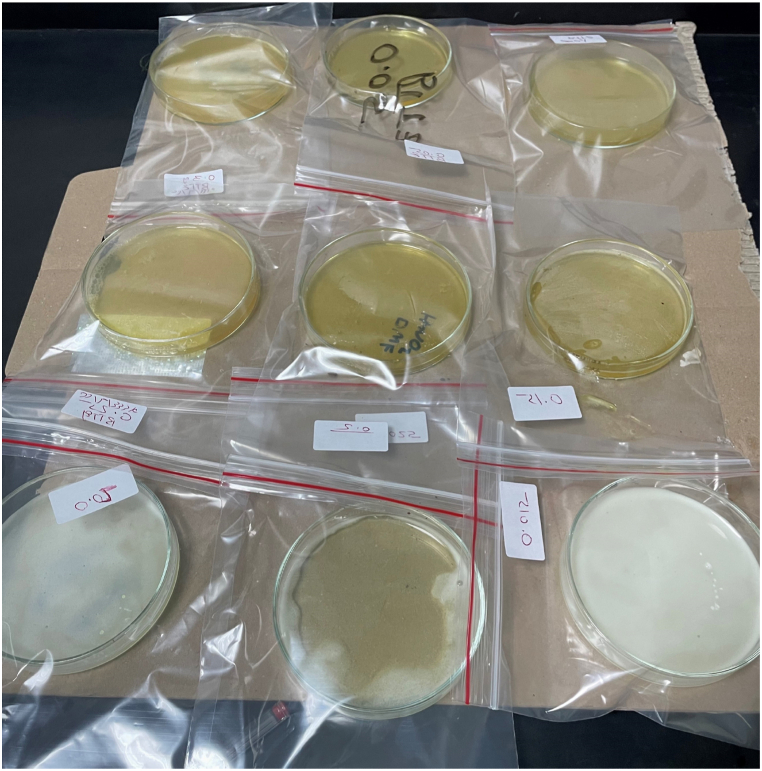
PVA/NBT film samples with different concentrations of NBT before irradiation.

**Fig. 12:**
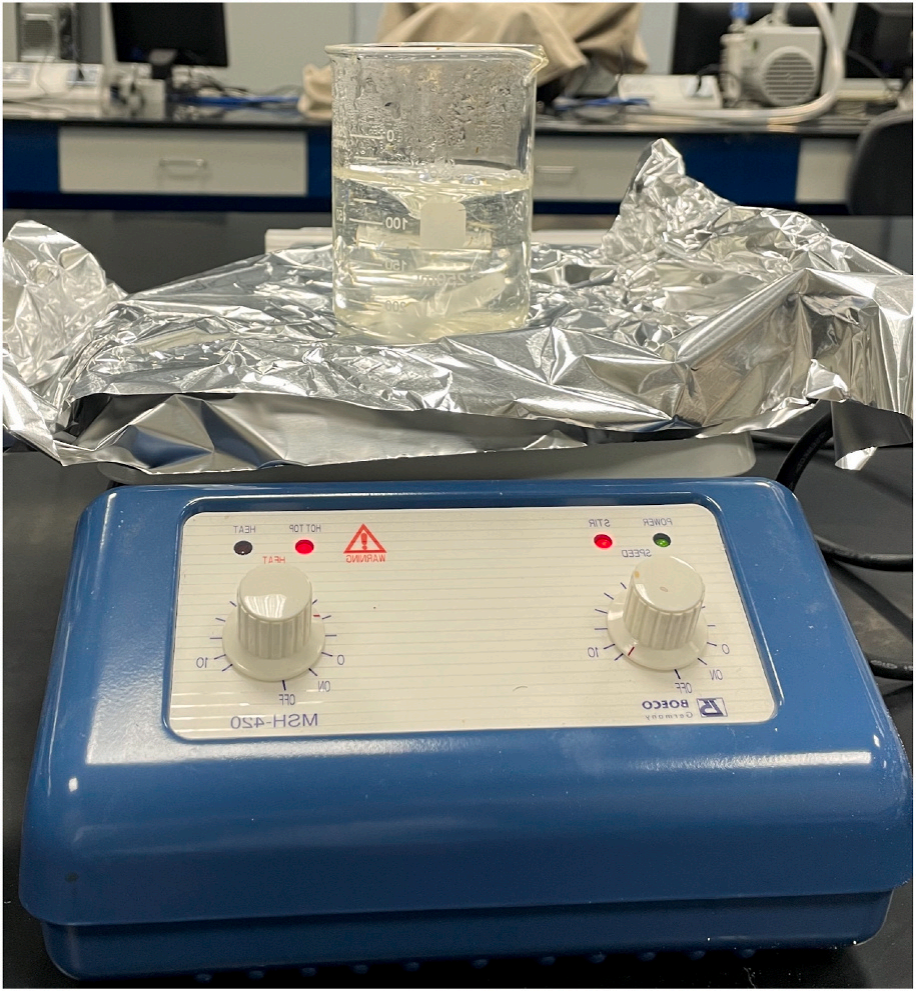
During the preparation of the PVA solution using the magnetic stirrer.

**Fig. 13:**
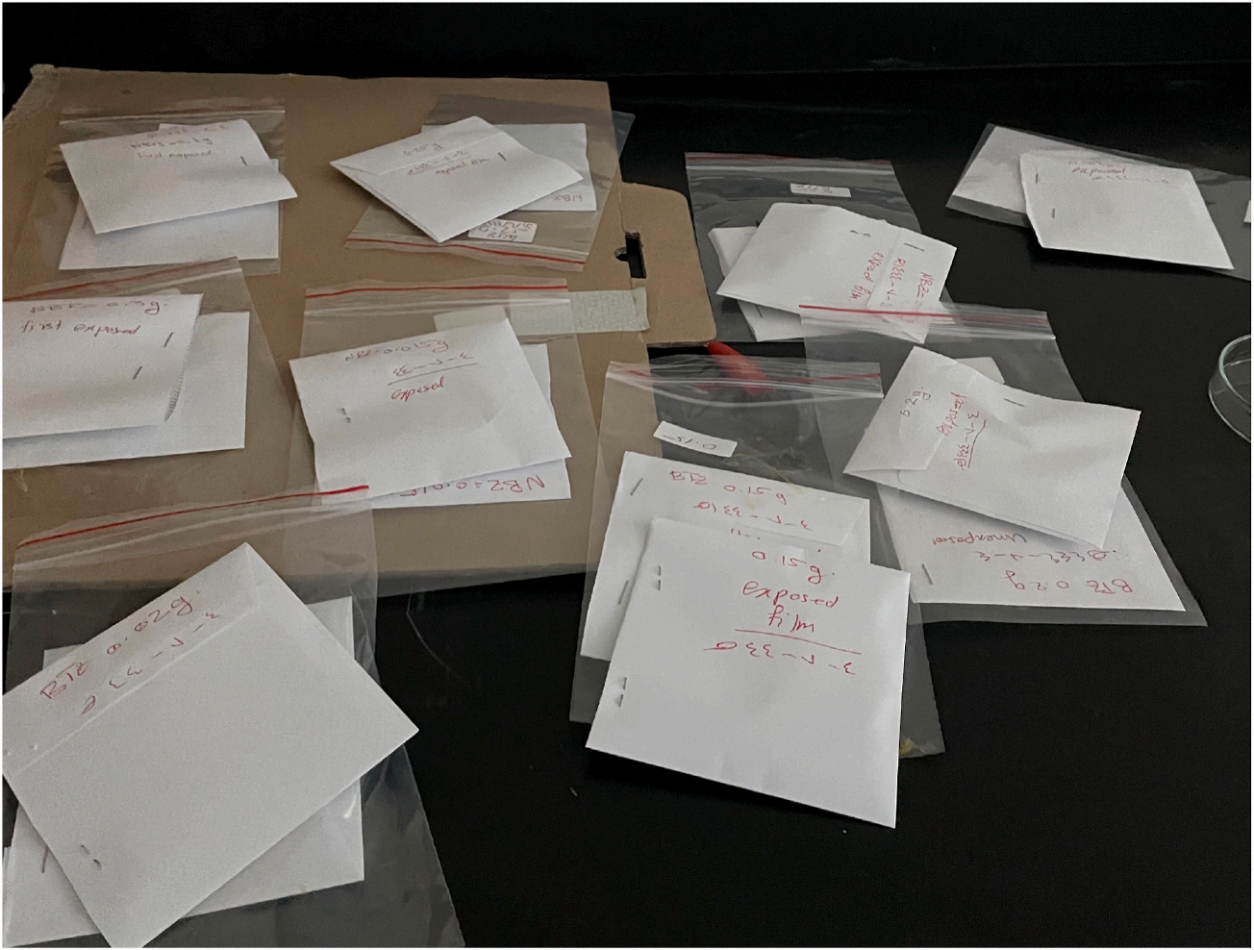
PVA/NBT films post-irradiation, preserved in airtight envelopes to shield them from light.

**Fig. 14:**
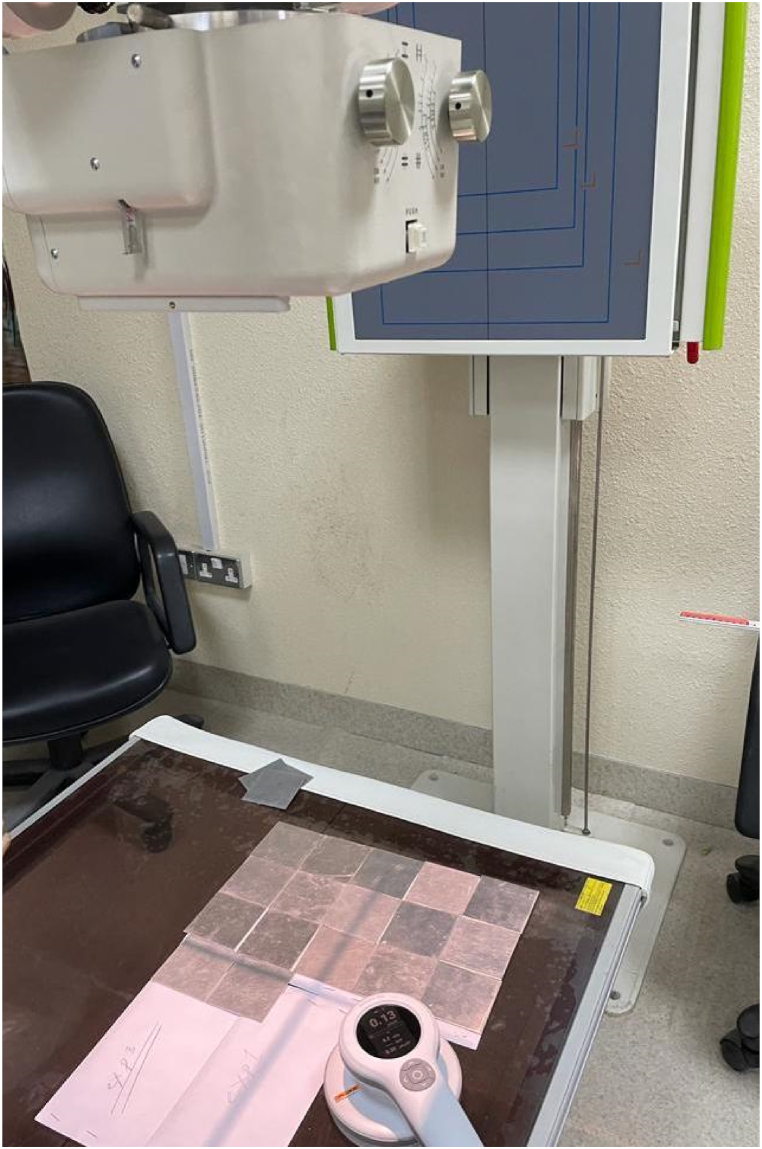
Films during irradiation by an X-ray machine.
